# Out of Shape During Stress: A Key Role for Auxin

**DOI:** 10.1016/j.tplants.2018.05.011

**Published:** 2018-09

**Authors:** Ruud A. Korver, Iko T. Koevoets, Christa Testerink

**Affiliations:** 1 University of Amsterdam, Plant Cell Biology, Swammerdam Institute for Life Sciences, 1090GE Amsterdam, The Netherlands; 2 Laboratory of Plant Physiology, 6708PB Wageningen University and Research, Wageningen, The Netherlands

**Keywords:** auxin, auxin transport, IAA homeostasis, abiotic stress, mathematical modeling, root phenotypic plasticity

## Abstract

In most abiotic stress conditions, including salinity and water deficit, the developmental plasticity of the plant root is regulated by the phytohormone auxin. Changes in auxin concentration are often attributed to changes in shoot-derived long-distance auxin flow. However, recent evidence suggests important contributions by short-distance auxin transport from local storage and local auxin biosynthesis, conjugation, and oxidation during abiotic stress. We discuss here current knowledge on long-distance auxin transport in stress responses, and subsequently debate how short-distance auxin transport and indole-3-acetic acid (IAA) metabolism play a role in influencing eventual auxin accumulation and signaling patterns. Our analysis stresses the importance of considering all these components together and highlights the use of mathematical modeling for predictions of plant physiological responses.

## Highlights

Recent findings in active auxin transport over organelle membranes and the influence of apoplastic and cytosolic pH on passive auxin flow into the cell highlight the importance of short-distance auxin transport for local auxin maxima in roots exposed to stress.

Based on previously published gene expression data, we have identified stress-induced expression patterns of IAA biosynthesis and conjugation genes in the root that could be relevant for IAA accumulation patterns. These results underscore the importance of incorporating IAA homeostasis into models predicting auxin levels and flow.

Recent advances in mathematical models involving auxin-related processes highlight the value of combining *in planta* and *in silico* experiments when unraveling complex hormonal changes in plants.

## Auxin on the Move During Stress

Drought and increasing salinity are abiotic stresses that cause major decreases in crop yield worldwide. Drought causes loss of crops through water deficit, whereas increasing soil salinity induces osmotic and ionic stress in the plants. Both abiotic stresses are a threat to the amount of arable land that is fit for our food production. Although the development of crops tolerant to these conditions has received attention [1], a greater research focus has been on generating biotic stress resistance and increasing the yield of edible parts of the plant. Now that we face a rapid deterioration of arable land, research on the tolerance to abiotic stresses has substantially increased. Phenotypic plasticity, including developmental modifications to root system architecture (RSA), is vital for tolerance to water deficiency and high soil salinity.

RSA and root growth rates during plant development under optimal conditions have been well studied, and it has long been established that these require the phytohormone **auxin** (see Glossary). Recently our fundamental understanding of root developmental plasticity during abiotic stress has markedly improved [2]. Unsurprisingly, auxin plays an important role during abiotic stress-induced changes in the root. Through the creation of local auxin maxima, cell elongation is locally inhibited and the emergence of lateral roots can be arrested. On the other hand, local auxin minima were found to be a signal that triggers the transition from cell division to cell differentiation in roots of arabidopsis (*Arabidopsis thaliana*) [3]. The different processes that together determine auxin-mediated regulation of growth and development during abiotic stress are auxin transport, biosynthesis, conjugation, perception, and signaling. Auxin transport has received much attention, and the role of polar auxin transport (PAT) by auxin carrier proteins during unstressed conditions and gravitropism has been well established 4, 5, 6. By contrast, the changes in PAT during abiotic stresses remain largely unknown. How changes in local auxin biosynthesis and **IAA conjugation** during abiotic stress affect root responses is another relatively young field of research. The integration of all these different aspects of auxin homeostasis is complicated because the many factors involved all influence each other and there is extensive crosstalk between auxin and other hormones. One promising solution to this problem is the rapidly emerging field of computational modeling of auxin processes in the plant root.

## Auxin Transport from Shoot to Root

**Figure 1:**
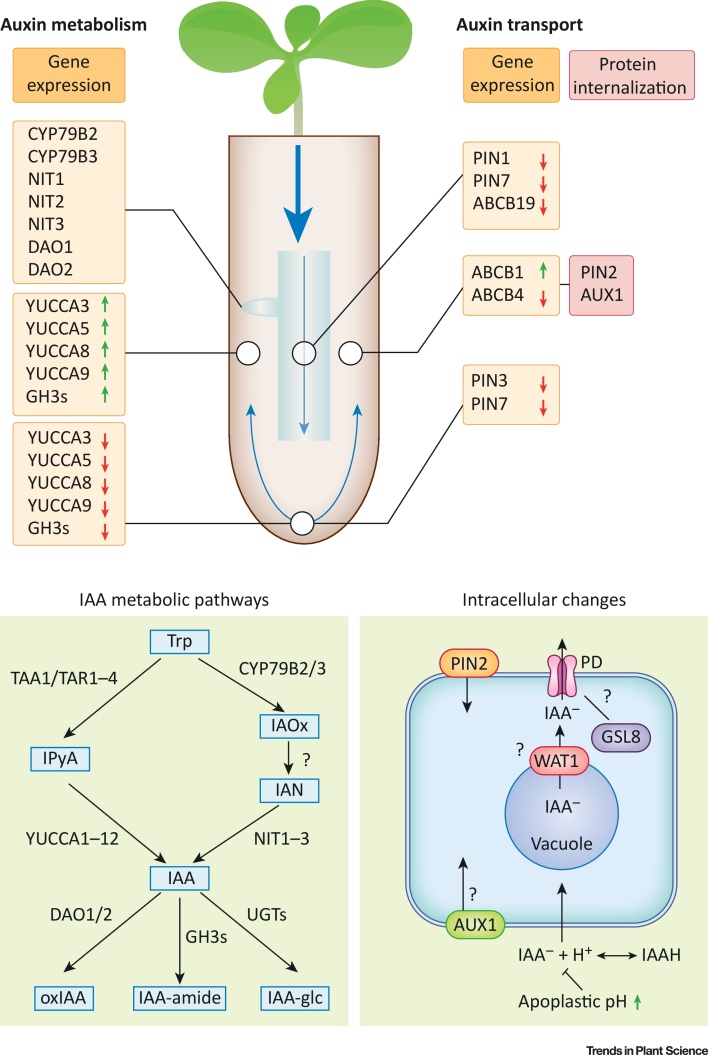
Schematic Overview of Salt Stress-Induced Changes in Auxin Biosynthesis, Conjugation, and Transport-Related Processes in the Arabidopsis Roots. (A) Indole-3-acetic acid (IAA) metabolism. The level of IAA is tightly regulated by IAA biosynthesis, conjugation, and degradation, together determining the IAA status of a cell. The lower panel shows part of the known IAA biosynthesis (21, 22 for a complete overview) and conjugation pathways and the known involved genes or gene families. Based on microarray data of two different studies (Table S1), we have identified gene expression patterns with promising possibilities for influencing eventual IAA accumulation patterns and signaling. For genes in the IAOx pathway we observe strong expression in zone 4 (Figure 2C), which is the zone containing primordia and lateral roots. All genes are also influenced by salt stress in a time-dependent matter (Table S1). We see a similar pattern for DAO1 and DAO2. Both DAO1 and CYP79B2-3 have been linked to lateral root development and show expression specifically underlying newly formed lateral roots. YUCCA 3, 5, 8, and 9, together with the GH3 family genes, show upregulation in the epidermis and cortex during salt stress, whereas they are downregulated in the columnella (Figure 2B). (B) Auxin transport. During salt stress, expression of the auxin efflux carriers PIN1, PIN7, and ABCB19 in the stele is downregulated. In the columnella, PIN3 and PIN7 are downregulated. In epidermal and cortical cells, PIN2 and ABCB4 auxin efflux carriers are downregulated whereas ABCB1 is slightly upregulated. In epidermal cells, on a cellular level, PIN2 is internalized and changes in AUX1 abundance at the plasma membrane during halotropism were observed, providing evidence that AUX1 is also internalized [9]. Putatively, the activity of the tonoplast-located WAT1 undergoes changes to alter intracellular auxin levels following a change in cytosolic pH. To create local auxin maxima, the passive flow of IAA^−^ molecules through the plasmodesmata (PD) might need to be blocked. This is putatively achieved through GSL8-mediated callose deposition. The apoplastic pH increase following salt exposure of the root potentially inhibits the passive influx of IAAH into the cell. Blue arrows depict auxin flow, grey boxes show up- (green arrow) or downregulation (red arrow) of genes during salt stress. Black boxes show plasma-membrane proteins that are internalized upon salt stress. Question marks show processes that influence local auxin concentrations but have not yet been proved to be involved in local auxin changes during abiotic stress. Abbreviations: IAA^−^, ionized IAA; IAA-glc, IAA-glucose; IAAH, protonated IAA; IAN, indole-3-acetonitrile; IAOx, indole-3-acetaldoxime; IPyA, indole-3-pyruvic acid; oxIAA, oxidized IAA (2-oxindole-3-acetic acid); PD, plasmodesma; Trp, tryptophan.

The main mechanism to maintain the ‘upside-down fountain’ of auxin flow in the root is PAT. Clarification of the role of long-distance shoot-derived auxin transport in abiotic stress has advanced our understanding of the changes in auxin carrier proteins and other proteins that influence auxin flow in the root (Figure 1). Internalization of the auxin efflux carrier PIN-formed 2 (PIN2) either during **halotropism** on the side of the root facing a higher salt concentration [7] or during osmotic stress treatments [8] has been shown. Subsequently, by combining *in planta* salt stress experiments with computational modeling, it was concluded that internalization of PIN2 is not sufficient to explain the alteration of auxin flow during halotropism [9], and changes in PIN-formed 1 (PIN1) and auxin transporter protein 1 (AUX1) were shown to co-facilitate the fast change of auxin flow. In addition to root growth, auxin efflux carriers are suggested to regulate meristem size during salt stress [10]. Reduced PIN1, PIN3, and PIN7 expression and auxin-resistant 3 (AXR3)/indole-3-acetic acid 17 (IAA17) stabilization during salt stress have been proposed to influence the root meristem size by increasing nitric oxide (NO) levels.

Another large family of auxin carriers influencing auxin flow in the root is the ABCB transporter family [11]. Recently, several studies have shown a role for ABCB transporters during salt stress. Of 22 different ABCB transporters in rice (*Oryza sativum*), the expression of 21 was found to change in response to salinity and drought 12, 13. Of the root-expressed ABCB auxin transporters, the expression of ABCB1 and 19 was slightly upregulated, whereas ABCB4 showed downregulation after 1 h of salt stress. Reduced **acropetal transport** of auxin was observed in an *abcb19* null mutant, whereas **basipetal transport** was unaltered [14].

Other genes, whose loss-of-function mutants were recently observed to exhibit altered auxin flow in the root, are putatively involved in abiotic stress tolerance. Mutants of interactor of synaptotagmin 1 (ROSY1-1) showed a decrease in basipetal auxin transport and exhibited increased salt tolerance, which was ascribed to the interaction between ROSY1-1 and synaptotagmin 1 (SYT1) [15]. Furthermore, zinc-induced facilitator-like 1 (ZIFL1), a major facilitator superfamily (MFS) transporter, regulates shootward auxin efflux in the root [16]. *zifl1* loss-of-function mutants were observed to have reduced PIN2 protein abundance in epidermal root cells after external application of IAA and had gravitropic bending defects.

## Auxin Transport from Close By

Changes in auxin transport between different intracellular compartments also influence the auxin that is available for the formation of local auxin maxima. Auxin located in the acidic vacuole, with a pH of 5.0 to 5.5, will tend to move towards the cytosol which has a pH of ∼7. During salt stress the cytosolic pH drops [17], thus theoretically reducing passive auxin efflux from the vacuole. Apoplastic pH is also suggested to be involved in passive auxin influx into the cells (Box 1).

Box 1 **Apoplastic pH and Auxin Movement**

Auxin transport is, in addition to active processes, dependent on passive movement of IAA into or between cells and movement through the apoplast. The concentration of auxin influences apoplastic pH, which in turn influences passive auxin transport into cells. In roots, high cellular auxin concentrations inhibit cell elongation, whereas in the shoot, in accordance with the acid growth theory, auxin causes cell elongation 66, 67.

The effect of auxin on apoplastic pH has been demonstrated by the addition of exogenous auxin to arabidopsis roots, which induced fast alkalization of the apoplast [68]. However, after prolonged exposure (8 h) the root apoplast becomes acidified. Similarly, initial alkalization of the apoplast followed by acidification after 19 h was found when endogenous auxin levels were elevated by induced expression of YUCCA6 [68]. Barbez and colleagues have demonstrated that cell-wall acidification triggers cell elongation in arabidopsis seedlings. Their data imply that endogenous auxin concentrations regulate apoplast acidification, which in turn regulates cell elongation. During exposure of roots to a salt gradient, auxin redistributes in the root 7, 69, suggesting that local alterations in apoplastic pH levels during salt stress might influence cell elongation. In addition, following 1 h root exposure to NaCl a transient increase in apoplastic pH was observed [17]. Following a 100 mM NaCl pulse apoplastic pH returned to control levels 1 h after the transient increase in apoplastic pH. The cytoplasmic pH decreased slightly upon NaCl exposure but did not recover. The changes in passive auxin transport as a result of this alteration in apoplast pH have not yet been studied *in vivo*. Nonetheless, a mathematical/computational model linking auxin and pH dynamics has been created [61]. The main conclusions from this model are that long-term auxin-induced apoplast acidification leads to increased auxin transport across the plasma membrane and significantly higher auxin concentrations in the cytoplasm. However, it has also been reported that the level of passive auxin influx would be negligible at low apoplastic pH (<5.7) in protoplasts [70]. This would mean that all changes in intracellular auxin levels take place through active auxin transport, and there is no role for passive auxin transport in pH-induced alteration of cell elongation during abiotic stress. *In vivo* measurements of cellular auxin influx and efflux during apoplast acidification will be necessary to elucidate the role of passive auxin transport across the plasma membrane.

Alt-text: Box 1

Isolated vacuoles from protoplasts lacking the tonoplast-located auxin carrier WAT1 (walls are thin 1) were found to accumulate significantly more radiolabeled auxin than wild-type vacuoles, indicating active transport of auxin from the vacuole to the cytoplasm by WAT1 [18]. Recently, an auxin transport facilitator family, located on the endoplasmic reticulum (ER), was identified. PIN-LIKES (PILS) proteins are believed to be involved in auxin homeostasis through auxin accumulation at the ER, in this way limiting the IAA available for nuclear auxin signaling. The change in available IAA alters the cellular sensitivity to auxin. Increased auxin export from *pils2/pils5* protoplast cells was also observed [19]. In addition, a *pils2* arabidopsis mutant, and even more so a *pils2/pils5* double mutant, showed significantly longer roots than the wild type and a higher lateral root density, suggesting PILS involvement in auxin-dependent root growth.

Other forms of passive auxin transport include movement without the interference of membranes. IAA is a small molecule and is therefore able to move freely through the plasmodesmata (PD) in its ionized form (IAA^−^). To restrict free cell-to-cell movement of IAA during auxin gradient formation, GLUCAN SYNTHASE-LIKE 8 (GSL8) induces an increase of plasmodesma-localized callose. This reduces the symplasmic permeability to maintain local auxin maxima [20]. GSL8 expression was found to be upregulated by adding exogenous IAA. Although these results demonstrate the importance of local auxin movement, the relation to abiotic stress remains to be elucidated.

## Controlling the Levels of Auxin

In addition to transport, auxin (IAA) levels are determined by biosynthesis and conjugation. Both processes have recently shown to be affected by abiotic stress in the root.

Although several IAA biosynthesis pathways has been described 21, 22, the indole-3-pyruvic acid (IPyA) pathway is considered to be responsible for most IAA biosynthesis in higher plants 23, 24. In addition, for the Brassicaceae family, the indole-3-acetaldoxime (IAOx) pathway has been increasingly identified to play a role during several stress responses 25, 26, 27, whereas the significance of the other pathways remains under debate and needs further research 21, 22. We focus here on the IPyA and IAOx pathways, and on how these pathways are modulated during abiotic stress.

**Figure 2:**
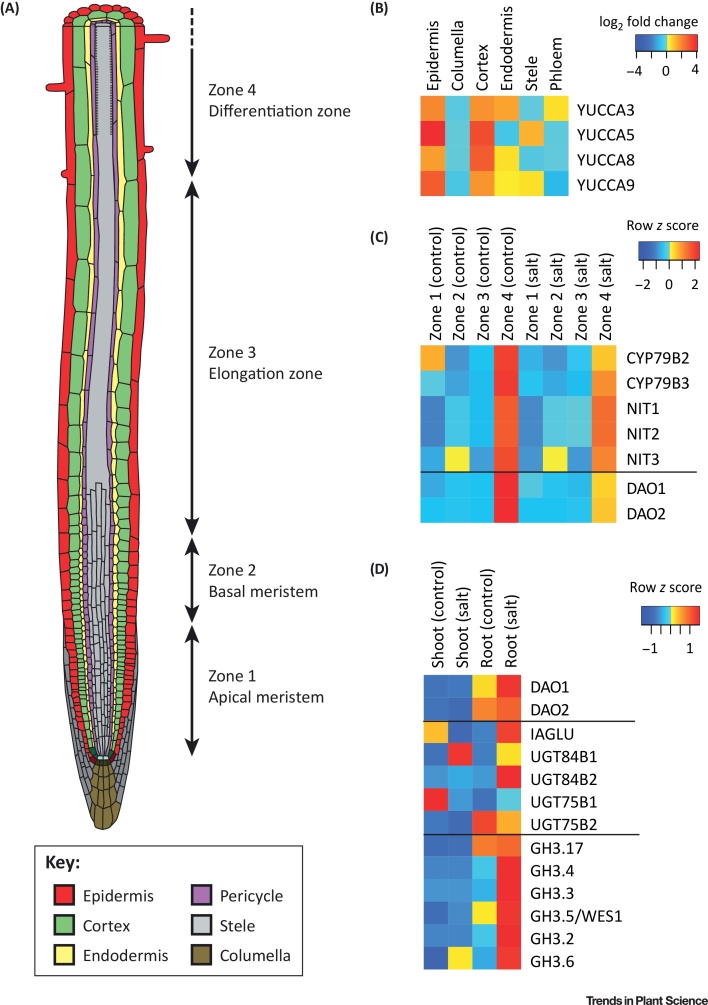
Illustration of Gene Expression of Auxin Homeostasis-Related Genes Showing Patterns That Could Be Relevant for Indole-3-Acetic acid (IAA) Accumulation Patterns in Abiotic Stress. (A) Heatmaps are based on microarray data of two different studies (Table S1) 37, 38. For tissue-specific and zone specific expression patterns, the root has been divided in different tissues and in four different root zones, as illustrated. Figure adapted, with permission, from Figshare (B. Peret, Primary and lateral root.ai; https://figshare.com/collections/Root_illustrations/3701038). (B) Heatmap of the relative expression (log_2_ fold change) upon salt stress of YUC3, 5, 8, and 9 in different root tissues. (C) Heatmap of the expression of genes in the indole-3-acetaldoxime (IAOx) pathway and oxidation in different root zones under control conditions and during salt stress. The heatmap is normalized per gene. (D) Heatmap of the expression of genes involved in IAA conjugation in the shoot and root under control conditions and during salt stress. The heatmap is normalized per gene.

The IPyA pathway (Figure 1) generates IAA via a two-step conversion from tryptophan, with IPyA as the intermediate 27, 28, 29. The family of YUCCA proteins governs the second step of the pathway [27]. Eleven YUCCA isoforms have been described in arabidopsis, and specific roles for several YUCCAs are slowly being elucidated. The YUCCAs can be divided into mainly root- or shoot-active proteins 30, 31. YUC3, 5, 7, 8, and 9 display distinct expression patterns in the root, whereas other YUCCAs show minor or no expression in the root [30]. This illustrates the specificity of different genes in this pathway for specific developmental processes. Although little research on the specificity of YUCCAs for different stresses has been carried out, several gene expression studies do indicate this specificity. For example, YUC2, 5, 8, and 9 show upregulation in plants experiencing shade [32], and knockout mutants of these quadruple YUCCA lack shade-induced hypocotyl elongation [33]. Several papers show that overexpression of the IPyA pathway leads to increased salt tolerance in several species 34, 35, 36. For example, it was shown in cucumber that specific YUCCAs are expressed at high and low temperature and in response to salinity [36]. In salinity, CsYUC10b is strongly upregulated and CsYUC10a and CsYUC11 are strongly downregulated, whereas overexpression of CsYUC11 leads to higher salinity tolerance [36]. For arabidopsis, the role of specific YUCCAs during salt stress is so far unknown. Analysis of previously published microarray data 37, 38 confirms the root specificity of YUC3, 5, 8, and 9 (Table S1 in the supplemental information online). Furthermore, tissue-specific microarray data indicate a shift from strong expression of YUCCAs in columnella under control conditions to strong expression in the epidermis and cortex during salt stress (Figures 1, 2A, and Table S1). Because expression of YUCCAs is low in the epidermis and cortex under control conditions, and thus auxin levels in these cells would mainly depend on transport, this is an interesting shift. Salt stress also has major effects on auxin transport, and therefore epidermal biosynthesis is likely to affect auxin distribution during stress and is expected to have consequences for growth responses in the root.

The IAOx pathway (Figure 1) is *Brassica*-specific and has mostly been described for its role in secondary metabolism, producing both indole glucosinolates and camalexin 27, 39, 40, 41. More recently, however, several papers have pointed out a possible involvement of this pathway in local IAA production, specifically under stress conditions 25, 26, 27, 42. Sugawara *et al*. showed that, when plants were fed ^13^C_6_-labeled IAOx, 40% of the ^13^C_6_ atoms was incorporated into IAA, confirming that IAA can be formed from IAOx [42]. ln 2002 Zhao *et al*. showed that a *cyp79b2 cyp79b3* double-knockout mutant showed reduced growth and reduced IAA production specifically under higher temperatures [27]. Recently, the same mutant was observed to have decreased lateral root growth during salt stress [25]. Whereas the IPyA pathway is mainly active in the differentiation and elongation zone of the main (and lateral) root, genes in the IAOx pathway are, in addition to expression in the QC, strongly expressed in cells underlying newly developing lateral roots and lateral root primordia [43]. In accordance, all genes described in this pathway show strong expression in the differentiation zone (Figure 2A,B and Table S1) and their expression during salt stress is strongly time-dependent (Table S1), suggesting a major role for this pathway in salt-regulated lateral root development in arabidopsis.

Levels of free IAA are tightly regulated by several conjugation and degradation processes (Figure 1) [44]. Although IAA itself is both the active and transported compound in plants, it has a very high turnover. *In planta*, the levels of IAA conjugates and catabolites correlated strongly with the levels of free IAA in both root and shoot, and high levels of conjugates and catabolites are present when high levels of free IAA occur 45, 46. Levels of IAA catabolites are in general higher than of free IAA [45]. The strict regulation of free IAA levels is nicely illustrated by analysis of mutants in specific conjugation pathways – reduced conjugation via one pathway often leads to increased conjugation via other pathways and only minor changes in free IAA levels [47].

Ester conjugation is mainly governed by several UDP-glucose transferases (UGTs). In Table S1 we have summarized the UGTs that are currently known to conjugate IAA, but this list is probably not complete. These enzymes respond differently to salt stress (Figure 2C and Table S1), which might point to specific roles for specific UGTs.

Enzymes of the GRETCHEN HAGEN 3 (GH3) family form amide conjugates of IAA [48]. All involved GH3s appear to be strongly expressed in roots and are upregulated upon salt stress (Figure 1C and Table S1). In addition, GH3.5/WES1 is induced by many abiotic and biotic stresses as well as by ABA treatment [49]. Interestingly, upregulation of GH3s upon salt stress seems to be specific to epidermis, comparable to upregulation of YUCCAs.

**IAA oxidation** is responsible for most IAA turnover and leads to degradation of the compound [50]. Plants contain 10–100-fold more oxIAA than IAA conjugates, showing that the oxidation pathway is very active [45]. Two DIOXYGENASE FOR AUXIN OXIDATION (DAO1 and DAO2) enzymes have been described to be responsible for the first step of oxidation 51, 52. DAO1 and 2 show strong expression in zone 4, similarly to genes in the IAOx pathway (Figure 2B and Table S1). Zhang *et al*. have shown that DAO1 is expressed in cells underlying newly developing lateral roots, and that the knockout has increased lateral root density [52], indicating a possible role for IAA oxidation in root responses to salt stress.

In addition to the main auxin, IAA, three other auxins have been described [53]. Of these, only indole-3-butyric acid (IBA) has been clearly shown to be of importance in (root) development [54]. For example, oscillations in IBA production determine pre-branch sites, cells that can potentially form lateral roots at later developmental stages 55, 56. Like IAA, IBA can be conjugated. In addition, it can be converted to IAA and possibly back again [57]. IBA and its conjugates are thereby also a possible storage form of IAA and can influence IAA homeostasis. Conjugation of IBA has been shown to affect salt tolerance because overexpression of UGT74E2, an IBA conjugating enzyme, leads to increased salt tolerance [58]. Whether IBA directly or indirectly – as a storage form of IAA – plays a role remains to be elucidated.

These examples show the importance of including IAA biosynthesis, conjugation, and degradation in the bigger picture of auxin homeostasis and accumulation because these processes may play major roles in the specific regulation of local auxin-mediated responses. However, the wide range of pathways involved in **IAA metabolism**, the enzymes involved, and their responses to salt stress shows the complexity of interpreting and predicting changes in auxin homeostasis. If we combine this complexity with the observed changes in auxin transport and auxin signaling, predicting the effect of changes in any of these steps can be daunting. More knowledge on the specific regulation of biosynthesis and conjugation enzymes, together with a more comprehensive view of all processes affecting auxin levels and response, will greatly improve our ability to predict the effects of changes in these different components. Incorporation of IAA metabolic pathways into models will also be a great leap forwards for predicting auxin accumulation patterns inside the plant because both processes, in addition to auxin transport, may modulate auxin levels.

## Understanding the Role of Auxin in Stress Responses Through Computational Modeling

Predicting the overall role and impact of auxin biosynthesis and short-distance transport compared to long-distance transport in the plant root during development and stress response is a complex task, despite improvements in the sensitivity of *in vivo* auxin reporters [59]. To integrate all components of the different processes that affect local auxin levels, the computational strength of mathematical modeling has proved to be helpful. Many different models concerning auxin or related processes have been created [60].

Although highly instrumental, the current root models have not yet incorporated all relevant parameters and are generally too static to accurately predict the magnitude of the influence of local auxin biosynthesis, conjugation, and short- and long-distance transport on the root stress response over longer time-periods. Interestingly, recent advances in root modeling show promising results to help to overcome these issues (Box 2). Nonetheless, many valuable insights concerning short-distance auxin transport have come from computational modeling. Until now most computer models have been either analytic one-cell models or describe overall changes in the whole root. Both have been shown to be informative in their own way. For the interplay between auxin and pH, Steinacher *et al*. predict the effect of auxin on intra- and extracellular pH and how this in turn affects auxin concentrations [61]. Their main findings point towards a role for auxin-activated proton pumps which alter proton fluxes, thus affecting auxin transport. By combining the chemiosmotic hypothesis of auxin transport with auxin-induced apoplastic acidification (AAA), they predict increased influx and efflux of auxin, resulting in higher cytosolic auxin concentrations. Another recent single-cell computational model involving lateral root emergence shows the value of computational modeling in local processes involving lateral roots. Mellor *et al*. show that the experimentally observed ‘all-or-nothing’ expression pattern of auxin transporter-like protein 3 (LAX3) may be explained by bistability that creates a genetic ‘switch’ [62]. The model was found to agree with the experimental data only when the exogenously added auxin was decreased over time, suggesting conjugation of auxin and its subsequent degradation. This observation nicely shows the added value of modeling.

Box 2 **Important Advances in Root Modeling**

To create a realistic root model for reliable predictions about changes in auxin flow and local auxin concentrations, several model characteristics need improvement. Two things that will help towards perfecting static 2D models of the root are a realistic root shape and known auxin feedback loops. Van den Berg *et al.* have shown that both root shape and auxin feedback are important to show experimentally observed changes in auxin flow during halotropism [9].

Another characteristic of the models that needs development is movement. While informative in short-term changes, predictions by static models become less realistic over time because they lack the changes in local auxin maxima that are needed for or caused by growth and bending. Interestingly, recently growing and bending root models have been realized. For example, a model describing a growing and bending root in which local changes in auxin concentrations can be predicted by changing the dynamics of PIN polarization under the influence of changing elastic fields due to root bending was made [71]. Similar to existing experimental data, the auxin concentration was highest at the maximum curvature in the root.

Comparable to growing versus static models, the 2D models that are being used now to make predictions of auxin levels are valuable; however, ideally we would want to use a 3D model. Owing to the high complexity, not many 3D root models have been attempted, and those that are available have yet to incorporate many factors. One such 3D model which aims to simulate lateral root emergence through LAX3 and PIN3 modeling has been published [72]. The 3D mathematical model incorporates LAX3 expression and auxin transport. It was found that, for the experimentally observed LAX3 spatial expression to be robust, auxin inducible activity of the PIN3 efflux carrier is required. Consecutive induction of LAX3 and PIN3 ensures stable LAX3 expression.

Alt-text: Box 2

An example of the relevance of incorporating auxin conjugation and degradation into auxin computational modeling is a mathematical model describing GH3-mediated auxin conjugation [63]. The model includes the upregulation of GH3 by auxin, thus regulating its own degradation, and positive feedback of auxin on the LAX3 influx carrier. The model predicts oscillation of GH3 and LAX3 mRNA levels after an extracellular auxin increase. In addition, the initial predicted increase of GH3 suggests a possible cellular mechanism to protect cell auxin homeostasis during changes in extracellular auxin levels.

To increase our understanding of long-distance auxin transport, Mitchison *et al*. showed in a single-cell model that combinations of changes in auxin carriers are necessary to mimic experimentally observed auxin flows [64]. Similarly, van den Berg *et al*. and Moore *et al*. have shown in a whole-root model the importance of changes in auxin influx carriers in addition to efflux carriers for modulating auxin flow 9, 65.

To date, a model incorporating the changes in auxin biosynthesis and interplay between the different synthesis pathways has yet to be constructed (see also Outstanding Questions). This follows logically from the fact that much about auxin biosynthesis remains unknown. However, for a full understanding of changes of auxin in the root during stress the incorporation of realistic changes in auxin synthesis will be necessary.

Nonetheless, multiple studies have now shown model predictions supporting experimental data and have provided suggestions for follow-up experiments. Although *in planta* experiments take significant time in the creation of tools to change single parameters, multiple parameters are easily changed in the models and putative regulators of auxin flow can be found. These regulators can then be verified by *in planta* experiments. Ideally, a growing and bending model incorporating as many factors as possible – different auxin carriers, hormonal feedback, mechanical feedback, pH changes, and the ability to simulate different stresses – would be useful to make predictions about growth rate and direction during abiotic stress. Furthermore, what modern models are still missing is the ability to show changes in RSA during stress. To elucidate the mechanisms behind different RSA strategies during different abiotic stresses, a model incorporating lateral root emergence and growth through the prediction of the local auxin concentrations is required. Again, such a complex model would need to take into account multiple factors dealing with local auxin maxima around lateral root primordia. Looking to the future of research on complex biological processes, such as the auxin machinery in the plant root, it becomes clear that the field of mathematical modeling will undoubtedly play a key role.

Outstanding Questions What is the contribution of shoot-derived auxin during stress-induced changes in auxin concentrations in the root?

How does local regulation of biosynthesis and conjugation contribute to auxin accumulation during abiotic stress?

What is the role of local auxin-regulated pH changes during changes in local auxin concentrations in response to stress?

## Glossary

**Acropetal transport**: transport from the base to the apex.
**Auxins**: plants contain multiple types of auxins, of which indole-3-acetic acid (IAA; also referred to as auxin) is the most abundant endogenous form which governs the majority of auxin-mediated effects in plants. Four other forms of endogenously produced auxins are indole-3-butyric acid (IBA), indole-3-propionic acid (IPA), 4-chloroindole-3-acetic acid (4-CI-IAA), and 2-phenylacetic acid (PAA).
**Basipetal transport**: transport from apex to the base.
**Halotropism**: directional growth of the main root away from a higher salt concentration when exposed to a salt gradient.
**IAA conjugation**: as part of IAA homeostasis, IAA can be conjugated, leading to inactive compounds of which some can be converted back to auxin and act as storage forms. The two main types of IAA conjugation are ester conjugation (IAA–sugar conjugate, reversible) and amide conjugation (IAA–amino acid conjugate, some are reversible).
**IAA metabolism**: all enzymatic pathways leading to the biosynthesis, conjugation, or degradation (oxidation) of IAA.
**IAA oxidation**: in addition to conjugation, IAA can also be oxidized. Oxidation always leads to degradation of auxin and is the major pathway responsible for fast IAA turnover.
